# The importance of quality of health campaign information for outcome evaluation. A case study from Guinea-Bissau and Bangladesh

**DOI:** 10.1016/j.jvacx.2024.100588

**Published:** 2024-11-18

**Authors:** Sebastian Nielsen, Sören Möller, Christine Stabell Benn, Peter Aaby

**Affiliations:** a Bandim Health Project, Open Patient Data Explorative Network, Department of Clinical Research, University of Southern Denmark, Denmark; b Bandim Health Project, Indepth Network, Apartado 861, Guinea-Bissau; c Open Patient Data Explorative Network, Department of Clinical Research, University of Southern Denmark, Denmark; d Danish Institute of Advanced Science, University of Southern Denmark, Denmark

**Keywords:** Campaign information, Oral polio vaccine, OPV, Vitamin A supplementation, Measles vaccine, Child mortality

## Abstract

**Background:**

Numerous national health intervention campaigns, e.g. supplementary immunization campaigns/activities (SIAs), have been conducted in low- and middle-income countries (LMIC) in the last decades. These campaigns are rarely evaluated for overall health outcomes. Information on campaigns is critical for evaluations. We investigated; 1) quality of campaign information sources and 2) implication of quality for outcome evaluations.

**Methods:**

We focused on three campaign types: oral polio vaccine (OPV), vitamin A supplementation (VAS) and measles vaccine (MV) campaigns in two case countries, for which “gold standard” information on campaigns collected regularly at Health and Demographic Surveillance Systems (HDSS) sites: Guinea-Bissau and Bangladesh. We compared the campaign information from HDSS with information from the World Health Organisation (WHO) and the Rotary Foundation (Rotary, only OPV campaigns). First, campaigns were matched and compared based on intervention type, date of campaign and target age group. Second, we assessed the implications of using various sources of campaign information on the estimated effect of OPV campaigns on all-cause under-3-year mortality in Cox proportional hazards regression models.

**Results:**

The proportion of matched OPV campaigns was highest between HDSS and Rotary. VAS campaigns (only information from HDSS and WHO) matched poorly. The estimated effect of OPV campaigns information on child mortality in Bangladesh went from being statistically significant (HR = 0.69 (0.52–0.90)) using HDSS campaign information to not being significant (HR = 0.93 (0.71–1.21) using WHO campaign information.

**Conclusion:**

Compared with the HDSS, Rotary had the best campaign information on the conduct of OPV campaigns, whereas the WHO quality of campaign information was low for both OPV and VAS. A low quality of campaign information may alter conclusions of health outcome evaluations. Reliable and precise information on campaigns is essential to assess their effects. Public and private campaign stakeholders should track campaign information meticulously and support that publicly data is available for researchers.

## 1 Introduction

Numerous national health intervention campaigns, e.g. supplementary immunization campaigns/activities (SIAs), have been conducted in low- and middle-income countries (LMIC) in the last decades. Oral polio vaccine (OPV) campaigns as part of the global efforts to eradicate wild poliovirus [1] has been one of the most common types of campaigns. The OPV campaigns have been orchestrated by the Global Polio Eradication Initiative (GPEI), a public-private partnership led by national governments with six partners – the World Health Organisation (WHO), Rotary International, the US Centers for Disease Control and Prevention (CDC), UNICEF, Gates Foundation and Gavi, the Vaccine Alliance. Other campaigns like those with vitamin A supplementation (VAS) and other vaccines have been coordinated by the WHO and UNICEF. For VAS, organisations like Helen Keller have also been involved.

Studies based on health and demographic surveillance system (HDSS) data from several low- and middle-income countries have shown that OPV campaigns have beneficial non-specific effects on child survival in settings where there has been no or very little wild poliovirus [[2], [3], [4], [5]]. The beneficial effects of OPV campaigns have primarily been found when OPV is administered alone and not with other interventions at the same time.

To conduct campaign evaluations and estimate their effects on health outcomes, e.g. all-cause child mortality, it is important to have reliable sources of campaign information. It is specifically necessary to know where the campaigns took place, which interventions were administered, the target age group and the exact dates of conduct.

Unfortunately, information on campaigns can be difficult to obtain at the country level. In some countries, HDSSs sites have collected reliable information on campaigns. In countries without that kind of data collection, the availability of information depends on whether the campaign information has been collected consistently by government or health authorities. Global stakeholders as a source of information on campaigns are crucial to support these data collections. The global sources for the campaign information includes the WHO, and for OPV more specifically the GPEI and Rotary that has sponsored numerous OPV campaigns.

In Guinea-Bissau and Bangladesh, information on campaigns has been collected at HDSS sites and the effect of OPV campaigns on child mortality has been analysed and published [2,3]. We have considered the HDSS campaign information “gold standard”. We set out to investigate the quality of different campaign information sources compared to the HDSS campaign information in Guinea-Bissau and Bangladesh. We focused on three frequent campaign types, OPV, VAS and MV campaigns and aimed to assess what the choice of source meant for the estimated effect of the OPV campaigns on child mortality.

## 2 Methods

### 2.1 Setting and study population

We focused on two case countries, Guinea-Bissau and Bangladesh. The HDSS sites included in these investigations are the Bandim Health Project (BHP, www.bandim.org) covering six urban districts in the capital Bissau of Guinea-Bissau in West Africa and following a population of around 100,000 people. The second HDSS is Chakaria (www.icddrb.org) covering 49 villages in south-eastern Bangladesh and following a population of around 120,000 people. More information on the cohorts and the analyses can be found in the previous publication [2,3]. The HDSS have formed the basis for analyses of the effect of OPV campaigns on child mortality. In this paper we have replicated the analyses using the different sources of campaign information.

### 2.2 Campaign information sources

In the two countries, we obtained campaign information from three different sources; 1) HDSS, 2) WHO and 3) Rotary (only OPV campaigns). We further requested access to campaign information from GPEI but was not allowed access to their data. GPEI referred to WHO. We also reached out to Hellen Keller Institute, a sponsor of numerous VAS campaigns in Bangladesh, but did not receive any reply to our inquiry. We did not manage to obtain any campaign information from governmental agencies in neither Guinea-Bissau nor Bangladesh. In Bangladesh the HDSS had contact with the local WHO office, which provided information on some specific campaigns conducted in Bangladesh, information that was used to supplement the HDSS campaign information [3].

#### 2.2.1 Exclusion criteria

Regardless of the campaign information source we excluded campaigns that had missing information on one or more of the three key features needed for the health intervention evaluation: type of intervention, date and age group. We also excluded campaigns that were registered as subnational (either denoted as “sub-national” or by assessing the size of the target population) and with no specified geographical location. Campaigns registered as being only planned but not conducted were also excluded (Supplementary Table 1).

#### 2.2.2 HDSS campaign information - the “gold standard”

Guinea-Bissau: At the Bandim Health Project HDSS, information on campaigns had been collected in real-time since the first national campaigns in 1998 [6]. We included the information on campaigns for the period from 2000 to 2014, corresponding to the period previously included in the analysis of the effect of OPV campaigns on under-3 years all-cause mortality [2] (Supplementary Table 2).

Bangladesh: In the Chakaria HDSS campaigns had not been recorded continuously during the period, therefore the HDSS campaign information was combined with campaign information from the local WHO office in Dhaka. We covered the period from 2001 to 2019, corresponding to the period included in the previous analysis of the OPV campaigns and under-3 years all-cause mortality in Chakaria [3] (Supplementary Table 2).

#### 2.2.3 WHO campaign information

The WHO campaign information was obtained through the international WHO headquarters in Geneva, Switzerland. We received the campaign information in four separate databases, one combined for the campaigns conducted before 2000 and one for each of the three types of vaccines conducted from 2000 (Supplementary Table 2). We excluded campaigns with missing information on intervention type (none), date (one VAS campaign in Guinea-Bissau and one MV campaign in Bangladesh) and age group (none) (Supplementary Table 1). We also excluded campaigns that were registered as subnational (three in Guinea-Bissau; VAS = 2, MV = 1 and eight in Bangladesh; VAS = 3, MV = 5) (Supplementary Table 1).

#### 2.2.4 Rotary OPV campaign information

We obtained information on OPV campaigns in Guinea-Bissau and Bangladesh from Rotary. The Rotary campaign information did not include information on target age group, but it was assumed that all campaigns targeted the recommended age group from 0 to 59 months of age (Supplementary Table 2). In Guinea-Bissau no Rotary OPV campaigns were excluded (OPV = 0). In Bangladesh five OPV campaigns were excluded as subnational (OPV = 5) (Supplementary Table 1).

### 2.3 Comparing sources of campaign information

We compared the information obtained from the different sources covering the period from 2000 to 2014 in Guinea-Bissau and 2001–2019 in Bangladesh with the HDSS campaign information as the reference “gold standard”. We investigated how well the information from WHO and Rotary matched these campaigns. It was considered a “full match” when the campaigns matched on all three key criteria:

1. Type of intervention administered (OPV, VAS or MV)
2. The first day of the campaign (DD/MM/YYYY)
3. Target age group (lower and upper age limit in months of age for the intervention)

The three criteria were chosen because they are important when analysing the overall effects of the campaigns on child mortality. We also considered “partial matches” allowing for one of the three criteria to differ (different intervention, date (within one month), or age group) between the sources.

### 2.4 Statistical analysis

#### 2.4.1 Assessment of data quality

We report the number of campaigns from the sources (WHO and Rotary) a) fully matched with the HDSS campaigns, b) partially matched with the HDSS campaigns, and c) extra campaigns in source not matched with the HDSS campaigns. We consider each intervention distributed as a separate campaign; e.g. if OPV and VAS were administered during the same period, we counted them as two separate campaigns.

#### 2.4.2 Impact of OPV on all-cause mortality depending on source of campaign information

We calculated hazard ratios (HRs) from multivariate Cox proportional hazards models comparing time after vs. before OPV campaigns as done in the previous analyses. We included the same covariates as previously [2,3]. Briefly, they include other campaigns (when available in the campaign information), time (calendar year) and age group (0–5, 6–11, 12–23, and 24–35 months of age) and follow the intention-to-treat principle that all children eligible to an intervention were assumed to have received it during the respective campaign intervention.

The proportional hazards assumptions were tested and assessed graphically using Schoenfeld residuals. Meta estimates and tests of heterogeneity of estimates were calculated using the *metan* command in Stata. We calculated two-sided 95 % confidence intervals and considered statistical significance at *p* < 0.05.

##### 2.4.2.1 Four different approaches to investigate the implications of campaign information for the estimation of the OPV mortality effect

First, we analysed the effect of OPV campaigns using only the information from WHO and Rotary, respectively, and compared with the estimate obtained with the HDSS data. In the analysis based on WHO data we adjusted for other campaigns using the WHO information on these campaigns. In the analysis based on Rotary data, we did not adjust for other campaigns since this information was not available from Rotary.

Second, we analysed the effect of OPV campaigns on mortality using OPV campaign information that a) fully matched, b) partially matched and c) extra campaigns in source not matched with the HDSS campaigns information. We adjusted for MV and VAS campaigns using the HDSS information.

Third, we analysed the effect of OPV campaigns on mortality with adjustment for MV and VAS campaigns using OPV campaign information from the HDSS but using the information on VAS campaigns from WHO. We also did the same using the information on MV campaigns from WHO. This was done to test the impact of lower-quality information on these adjustment factors for the estimation of the OPV effect, and was of particular interest for VAS, since VAS campaign misclassification could affect whether an OPV campaigns had been administered alone or as an OPV + VAS campaign.

Fourth, we investigated linear relationships (y = a∙x + b) between the proportion of follow-up time not matched after campaigns (x) and the deviation (y) in the resulting HRs from the analysis of the OPV campaigns using the original HDSS campaigns (the constant term in the linear Eq. (b) was assumed to be zero, as completely matched follow-up time was expected to yield no deviation in the HRs). As many comparisons of campaign information were partly overlapping, we included only the results from fully matched campaigns between sources and additional campaigns only available in the source.

All analyses were done using Stata version 17 and 18.

## 3 Results

### 3.1 Number of campaigns conducted

**Fig. 1:**
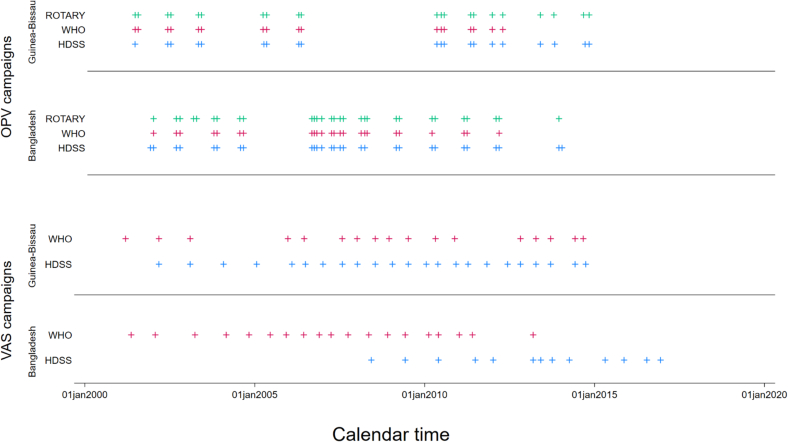
Overview of oral polio vaccination (OPV) and vitamin A supplementation (VAS) campaigns conducted in Guinea-Bissau and Bangladesh by campaign information source over calendar time. Each “+” represents a national campaign.

*Guinea-Bissau:* Between 2000 and 2014, the BHP HDSS had registered 46 campaigns of OPV (*n* = 20), VAS (*n* = 23) or MV (*n* = 3) that had been conducted among eligible children under 3 years of age; this information had been used for the analyses of the effect of OPV campaigns on child mortality [2]. The WHO had information on 37 campaigns (17, 17 and 3 respectively for OPV, VAS and MV) conducted in Bissau during the same period. (Table 1, Table 2 and Supplementary Table 3) Rotary had information on 21 OPV campaigns conducted (Table 2). The distribution over calendar time appeared similar for the OPV campaigns, while the VAS campaigns from WHO appeared to be incomplete compared with the HDSS (Fig. 1).

**Table 1:**
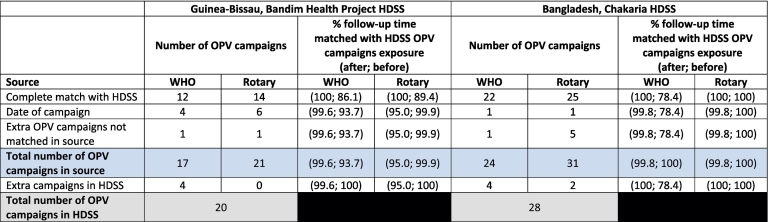
Overview of oral polio vaccination (OPV) campaign information from three different sources: health and demographic surveillance system (HDSS), the World Health Organisation (WHO) and the Rotary Foundation (Rotary). “Gold standard” OPV campaign information is from the HDSS. Number of fully matched, partially matched and extra additional non-matched OPV campaigns.

**Table 2:**
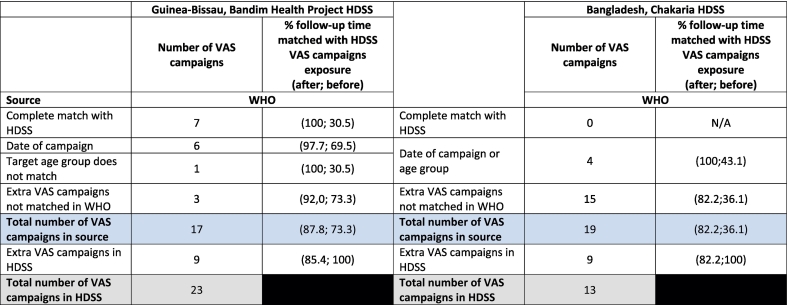
Overview of vitamin A supplementation (VAS) campaign information from two different sources: health and demographic surveillance system (HDSS) and the World Health Organisation (WHO). “Gold standard” VAS campaign information is from the HDSS. Number of fully matched, partially matched and extra additional non-matched VAS campaigns.

*Bangladesh:* Between 2001 and 2019, the Chakaria HDSS had registered 44 campaigns with OPV (*n* = 28), VAS (*n* = 13) and MV (*n* = 3). The WHO had information of 47 campaigns (24, 19 and 4 respectively for OPV, VAS and MV) in their campaign information (Table 1, Table 2 and Supplementary Table 3). Rotary had information on 31 OPV campaigns (Table 1).

The distribution over calendar time appeared similar for the OPV campaigns, but there were marked differences for the VAS where WHO had reported numerous campaigns before 2010, the HDSS only had a couple, while the opposite was observed for campaigns conducted from 2010 (Fig. 1).

### 3.2 Number of campaigns matched

#### 3.2.1 OPV campaigns

*Guinea-Bissau:* Among the 20 OPV campaigns conducted in the HDSS campaign information it was possible to fully match 60 % (*n* = 12) with the WHO campaign information on all three criteria. Further 20 % (*n* = 4) could be partially matched with less than one month difference in the date. There was one additional non-matched OPV campaign in the WHO data and four additional non-matched campaigns in the HDSS information. Rotary OPV campaign information could fully match 70 % (*n* = 14) of the HDSS OPV campaigns. Rotary also had further 30 % (*n* = 6) campaigns that could be partially matched with slightly different dates. Additionally, one campaign from Rotary and zero campaigns from the HDSS were not matched (Table 1).

*Bangladesh:* Among the 28 OPV campaigns conducted according to HDSS data it was possible to fully match 79 % (*n* = 22) with the WHO campaign information on all three criteria. Further 4 % (*n* = 1) could be partially matched with different dates (n = 1). There was one additional non-matched OPV campaign in the WHO data and four additional non-matched campaigns in the HDSS campaign information. Rotary OPV campaign information could fully match 89 % (*n* = 25) of the HDSS OPV campaigns. Rotary also had further 4 % (n = 1) campaigns that could be partially matched with slightly different dates. Additionally, five campaigns from Rotary and two campaigns from the HDSS were not matched (Table 1).

#### 3.2.2 VAS campaigns

*Guinea-Bissau:* Among the 23 VAS campaigns which were reported by the HDSS, it was possible to match 30 % (*n* = 7) completely with the WHO VAS campaign information. Additionally, 30 % (n = 7) were matched partially. WHO campaign information had reported three additional VAS campaigns and the HDSS had reported nine additional VAS campaigns (Table 2).

*Bangladesh:* Among the 13 VAS campaigns reported by the HDSS, it was not possible to completely match any with WHO VAS campaigns. Including partial matches, 31 % (*n* = 4) were matched with WHO campaigns. However, WHO reported additionally 15 VAS campaigns, while the HDSS had nine additional non-matched VAS campaigns (Table 2).

#### 3.2.3 MV campaigns

*Guinea-Bissau:* Three MV campaigns had been reported by the HDSS and they were all matched completely by the WHO MV campaigns information. There was no additional MV campaign in the WHO campaign data (Supplementary Table 3).

*Bangladesh:* Among the three MV campaigns reported by the HDSS, 67 % (*n* = 2) were matched completely with the WHO campaign information. There were no partial matches. The WHO had an additional two non-matched MV campaigns, while the HDSS information had one additional campaign (Supplementary Table 3).

### 3.3 Analysis of OPV campaigns and child mortality

*Guinea-Bissau:* When time after vs. before OPV campaigns were analysed in Cox models using only campaign information from the HDSS, the full model including all other campaign interventions yielded a HR of 0.75 (95 % CI: 0.67–0.85)(as previously reported, Table 3). When using OPV campaign information from WHO as the only source (adjusting for the other campaigns using HDSS data), the HR was 0.79 (0.71–0.87). Rotary OPV campaign information could only be analysed without adjustment for other campaigns, here the HR was 0.76 (0.68–0.84)(Table 3).

**Table 3:** Analysis of oral polio vaccination (OPV) campaigns by source of campaign information. Cox proportional hazards models comparing time after vs. before OPV campaigns yielding hazard ratios (HRs) and 95 % confidence interval (95 % CI).

Campaign information source	Estimate of the HR of time after OPV vs. before OPV campaigns (95 % CI) #1	Estimate of the HR of time after OPV vs. before OPV campaigns (95 % CI) #2
Guinea-Bissau, Bandim Health Project HDSS		
WHO #3	0.78 (0.70–0.87)	0.79 (0.71–0.88)
Rotary #4	0.76 (0.68–0.84)	N/A
HDSS	0.75 (0.68–0.83)	0.75 (0.67–0.85)
Bangladesh, Chakaria HDSS		
WHO #3	0.92 (0.71–1.21)	0.93 (0.71–1.21) #5
Rotary #4	0.81 (0.65–1.02)	N/A
HDSS	0.78 (0.62–0.98)	0.69 (0.52–0.90) #5
Combined estimate		
WHO #3	0.80 (0.71–0.88)	0.81 (0.72–0.89) #6
Rotary #4	0.77 (0.69–0.84)	N/A
HDSS	0.75 (0.69–0.82)	0.74 (0.66–0.82) #6

#1 Adjusting for age (underlying time) and year*age group.

#2 Adjusting for age (underlying time), year*age group and other campaign interventions. In HDSS, one additional H1N1 vaccination campaign (6–59 months of age) was conducted from the 14th of October 2010.

#3 Information on OPV, vitamin A supplementation (VAS) and measles vaccination (MV) campaigns.

#4 Information on OPV campaigns only.

#5 p-value for test of homogeneity of combined estimates = 0.13.

#6 *p*-value for test of homogeneity of combined estimates = 0.24.

*Bangladesh:* In the previous reported analysis using HDSS campaign information, the full model including all campaign interventions had yielded a HR of 0.69 (0.52–0.90)(as previously reported, Table 3). Using the OPV campaign information from the WHO yielded a non-significant HR of 0.93 (0.71–1.21) (*p*-value for test of heterogeneity, *p* = 0.13). Using Rotary OPV campaign information, the HR was 0.81 (0.65–1.02) (Table 3).

#### 3.3.1 Combined analysis by source

When combining the estimates from Guinea-Bissau and Bangladesh, the HDSS campaign information yielded a meta estimate of 0.74 (0.66–0.82). The analyses based on WHO campaigns yielded a HR of 0.81 (0.72–0.89). Both combined results suggest a significant beneficial impact of OPV campaigns. However, using the WHO campaign information suggested a less beneficial effect, though the difference was not significant (*p* = 0.24) (Table 3).

#### 3.3.2 Proportion of matched follow-up time and deviation in HR

When analysing the effect of using different OPV campaign information sources and studying the association between the proportion of time after OPV campaigns matched and the deviation in the estimated effect of OPV vaccine, a linear relationship was a good fit (R-squared = 0.91), y = 0.008∙x, where y is the deviation in HR and x the proportion of time after OPV campaigns matched. Since VAS and MV campaigns only included comparison of HDSS with WHO campaign information, only four data points were available for the analysis (it was eight for OPV). However, there was a trend that poor match of after VAS campaigns follow-up time was associated with an increased deviation, y = 0.001∙x (R-squared = 0.73). There was no trend seen for MV campaign information (Supplementary fig. 1).

## 4 Discussion

### 4.1 Main findings

“Gold standard” campaign information collected by the HDSSs in Guinea-Bissau and Bangladesh differed from the information provided by WHO and Rotary. The Rotary OPV campaign information matched the HDSS information better than the WHO OPV campaign information. Rotary only had information on OPV campaigns. VAS campaign information from the WHO matched poorly with HDSS campaign information in both countries. MV campaigns were fewer but matched generally better than VAS.

When evaluating the impact of OPV campaign information sources on their effects on all-cause mortality, we found that in Guinea-Bissau the effect of the OPV campaigns did not differ. In Bangladesh the effect went from being statistically significant, HR = 0.69 (0.52–0.90) with the HDSS campaign information to reporting no effect OPV campaigns on all-cause child mortality using the WHO campaign information, HR = 0.93 (0.71–1.21) (*p*-value for heterogeneity = 0.13). The impact of poor VAS campaign information, which was only included as an adjustment factor, did not have large implications for the estimated OPV campaign effect on child mortality.

### 4.2 Strengths and limitations

We only considered the information on the three main campaign interventions, OPV, VAS and MV, conducted in the last decades in Guinea-Bissau and Bangladesh. Other intervention campaigns and mass distribution activities have also been conducted, and could potentially be important to consider, e.g. mosquito net distributions yellow fever and cholera vaccine [7], [8], [9].

### 4.3 Interpretation

The Guinean HDSS collected campaign information continuously, often trying to accompany the vaccination team to collect individual information on who received the intervention and who did not; however, with the numerous campaigns this turned out to be too costly to collect individual information. Initially, there was not such a big focus on collecting campaign information and it is very likely that the one extra OPV campaign reported by both WHO and Rotary in November 2000, had been missed by the HDSS. Both WHO and Rotary reported this campaign, and it fits the original pattern of having two OPV campaigns with one month interval and the second one also including VAS. In Bangladesh, the collection of campaign information had been less intense and was therefore supplemented with information from the local WHO office. In both Guinea-Bissau and Bangladesh the same researchers have been involved throughout the respective study periods. In contrast, the data from WHO and Rotary has most likely been collected by different people in different periods. It may have been based on information of planned and/or already conducted campaigns. Delays and alterations in campaign planning has not been uncommon in Guinea-Bissau for a variety of local reasons and such changes and alterations may not have been returned to WHO or Rotary, probably leading to many of the partial matches. The campaign information by the WHO on the three different interventions were provided in four separate databases. This may also indicate that they may have been collected and managed by different people over time. MV campaigns were much less frequent than both OPV and VAS campaigns, but most of the MV campaigns were matched with all three criteria, 83 % (5/6). This could indicate that the MV campaign database may have had better and more reliable information compared to the VAS and OPV campaign databases. The MV campaign database is also the only one of the databases that is publicly available online [10].

### 4.4 Comparisons with other studies

No other study that we know of has investigated the quality of campaign information collected by HDSS with the information collected global stakeholders such as WHO and Rotary. However, other researchers have noted that the information on campaigns collected by the individual countries is potentially sparse and not easily accessible [11].

### 4.5 Implications

This study shows that information on campaigns collected by the global WHO office and Rotary differ from the information collected by the HDSS in Guinea-Bissau and Bangladesh. The implications of this variation of campaign information changed the results of analysis of the impact of OPV campaigns on child survival in Bangladesh when using the WHO campaigns information. Thus, though the trend overall remained similar, with a beneficial non-specific effect of OPV in a combined meta estimate from both HDSS, the beneficial effect in Bangladesh disappeared when only using the campaign information from WHO. It is therefore crucial that the WHO's global campaign data is used with great caution and preferably supported by other sources, e.g. Rotary for the OPV campaign information. In Bangladesh the information provided by the local WHO office in Dhaka also differed from the global WHO office. It is incomprehensible that the GPEI does not allow researchers to access their data.

## 5 Conclusion

The present study reveals that the information on national campaigns collected by the international stakeholders is of poor quality compared with the HDSS data collection and with the data collected by a campaign sponsor, Rotary. We furthermore showed that poor quality data may alter conclusions when used to evaluate campaign effects on health outcomes. It is therefore crucial that information on health intervention campaigns is collected meticulously. Public and private stakeholders alike should be motivated to keep track of health interventions to ensure that we will be able to evaluate their effects in the current and future contexts. Furthermore, such data should always be made publicly available for researchers.

## Funding

The work on non-specific effects of vaccines has been supported by the Danish Council for Development Research, Ministry of Foreign Affairs, Denmark [grant number 104.Dan.8.f.], Novo Nordisk Foundation and European Union FP7 support for OPTIMUNISE (grant: Health-F3–2011-261375).

## CRediT authorship contribution statement

**Sebastian Nielsen:** Conceptualization, Data curation, Formal analysis, Investigation, Methodology, Software, Validation, Visualization, Writing – original draft, Writing – review & editing. **Sören Möller:** Investigation, Methodology, Validation, Writing – review & editing. **Christine Stabell Benn:** Data curation, Formal analysis, Investigation, Methodology, Writing – review & editing. **Peter Aaby:** Conceptualization, Investigation, Methodology, Writing – review & editing.

## Declaration of competing interest

The authors declare that they have no known competing financial interests or personal relationships that could have appeared to influence the work reported in this paper.

## Data Availability

Data will be made available on request.

## Appendix A Supplementary data

Supplementary material Image 1
