# Supplementary material for: The importance of quality of health campaign information for outcome evaluation. A case study from Guinea-Bissau and Bangladesh
Source: Vaccine X. 2024 Nov 18;21:100588. doi: 10.1016/j.jvacx.2024.100588 (PMC11616566; doi:10.1016/j.jvacx.2024.100588)
Supplement: Supplementary file 1 — Supplementary material [file mmc1.docx]

**Supplementary Table 1. Overview of all excluded campaigns reported by WHO and Rotary in Guinea-Bissau and Bangladesh.**

| **Guinea-Bissau** | | | | | | |
| --- | --- | --- | --- | --- | --- | --- |
| **Campaign date**  **(DD-MM-YYYY)** | **Age group (in months)** | | | **Source** | | **Exclusion**  **criterion** |
|  | **OPV** | **VAS** | **MV** | **WHO** | **Rotary #1** |  |
| 01-05-2003 |  | 6-35 |  | X |  | Subnational |
|  |  |  | 0-11 | X |  | Subnational |
| 01-11-2004 |  | 6-35 |  | X |  | Subnational |
| ??-??-2006 |  | 6-35 |  | X |  | Missing Date |

| **Bangladesh** | | | | | | |
| --- | --- | --- | --- | --- | --- | --- |
| **Campaign date**  **(DD-MM-YYYY)** | **Age group (in months)** | | | **Source** | | **Exclusion**  **criterion** |
|  | **OPV** | **VAS** | **MV** | **WHO** | **Rotary #1** |  |
| 26-08-2001 | 0-35 |  |  |  | X | Subnational |
|  |  |  | 9-35 | X |  | Subnational |
| 10-08-2002 |  | 12-35 |  | X |  | Subnational |
| 03-09-2005 |  |  | 9-35 | X |  | Subnational |
| 14-10-2006 | 0-35 |  |  | X | X | Subnational |
| 20-05-2007 | 0-35 |  |  | X | X | Subnational |
| 01-07-2007 | 0-35 |  |  | X | X | Subnational |
| 07-01-2012 |  | 6-35 |  | X |  | Subnational |
| 02-06-2012 |  | 6-35 |  | X |  | Subnational |
| 11-12-2016 |  |  | 9-35 | X |  | Subnational |
| 29-04-2017 |  |  | 9-35 | X |  | Subnational |
| 16-09-2017 |  |  | 6-35 | X |  | Subnational |
| 27-01-2018 | 0-35 |  |  |  | X | Subnational |
| ??-??-???? |  |  | 9-35 |  |  | Misssing Date |

#1 Rotary only had information on OPV campaigns. Grey shading marks campaigns for other intervention types than OPV for which Rotary had no information.

**Supplementary Table 2. Overview of all campaigns reported in Guinea-Bissau and Chakaria by both the HDSSs and WHO. Showing first campaign date, intervention type, age group and source of campaign information and deviations in the WHO and Rotary information when partially matched. OPV campaigns marked in blue.**

| **Guinea-Bissau: The Bandim Health Project HDSS** | | | | | | | |
| --- | --- | --- | --- | --- | --- | --- | --- |
| **Campaign date**  **(DD-MM-YYYY)** | **Age group (in months)** | | | **Source** | | | **Deviations from**  **HDSS campaign information**  **Criterion: information (source)** |
|  | **OPV** | **VAS** | **MV** | **HDSS** | **WHO** | **Rotary #1** |  |
| 15-10-2000 | 0-35 |  |  | X | X | X | Date of month: 16-10-2000 (WHO, Rotary) |
| 20-11-2000 | 0-35 |  |  |  | X | X |  |
|  |  | 6-35 |  |  | X |  |  |
| 22-10-2001 | 0-35 |  |  | X | X | X |  |
| 26-11-2001 | 0-35 |  |  | X | X | X |  |
|  |  | 6-35 |  | X | X |  | Age group: 9-35 months (WHO) |
| 05-10-2002 | 0-35 |  |  | X | X | X |  |
| 09-11-2002 | 0-35 |  |  | X | X | X |  |
|  |  | 6-35 |  | X | X |  |  |
| 15-11-2003 |  | 6-35 |  | X |  |  |  |
| 18-10-2004 | 0-35 |  |  | X | X | X | Date of month: 08-10-2004 (WHO, Rotary) |
| 18-11-2004 | 0-35 |  |  | X | X | X |  |
|  |  | 6-35 |  | X |  |  |  |
| 01-11-2005 |  | 6-35 |  |  | X |  |  |
| 18-11-2005 | 0-35 |  |  | X | X | X |  |
| 16-12-2005 | 0-35 |  |  | X | X | X |  |
|  |  | 6-35 |  | X |  |  |  |
| 15-05-2006 |  | 6-35 |  | X | X |  | Date of month: 01-05-2006 (WHO) |
|  |  |  | 6-35 | X | X |  |  |
| 26-11-2006 |  | 6-35 |  | X |  |  |  |
| 30-06-2007 |  | 6-35 |  | X | X |  | Date of month: 28-06-2007 (WHO) |
| 14-12-2007 |  | 6-35 |  | X | X |  | Date of month: 11-12-2007 (WHO) |
| 30-06-2008 |  | 6-35 |  | X | X |  |  |
| 01-12-2008 |  | 6-35 |  |  | X |  |  |
| 07-01-2009 |  | 6-35 |  | X |  |  |  |
| 03-07-2009 |  | 6-35 |  | X | X |  |  |
|  |  |  | 9-35 | X | X |  |  |
| 19-01-2010 |  | 6-35 |  | X |  |  |  |
| 06-03-2010 | 0-35 |  |  | X | X | X |  |
| 23-04-2010 | 0-35 |  |  | X | X | X | Date of month: 24-04-2010 (WHO, Rotary) |
| 28-05-2010 | 0-35 |  |  | X | X | X |  |
|  |  | 6-35 |  | X | X |  | Date of month: 01-05-2010 (WHO) |
| 16-12-2010 |  | 6-35 |  | X | X |  | Date of month: 01-12-2010 (WHO) |
| 25-03-2011 | 0-35 |  |  | X | X | X |  |
| 29-04-2011 | 0-35 |  |  | X | X | X | Date of month: 28-04-2011 (Rotary) |
|  |  | 6-35 |  | X |  |  |  |
| 25-11-2011 | 0-35 |  |  | X | X | X |  |
|  |  | 6-35 |  | X |  |  |  |
| 23-03-2012 | 0-35 |  |  | X | X | X |  |
| 12-07-2012 |  | 6-35 |  | X |  |  |  |
| 02-12-2012 |  | 6-35 |  | X | X |  |  |
|  |  |  | 9-35 | X | X |  |  |
| 24-05-2013 | 0-35 |  |  | X |  | X |  |
|  |  | 6-35 |  | X | X |  |  |
| 03-11-2013 | 0-35 |  |  | X |  | X | Date of month: 25-10-2013 (Rotary) |
|  |  | 6-35 |  | X | X |  |  |
| 02-08-2014 |  | 6-35 |  | X | X |  |  |
| 14-10-2014 | 0-35 |  |  | X |  | X | Date of month: 27-09-2014 (Rotary) |
| 29-11-2014 | 0-35 |  |  | X |  | X |  |
|  |  | 6-35 |  | X | X |  | Date of month: 01-11-2014 (WHO) |

| **Bangladesh: The Chakaria HDSS** | | | | | | | |
| --- | --- | --- | --- | --- | --- | --- | --- |
| **Campaign date**  **(DD-MM-YYYY)** | **Age group (in months)** | | | **Source** | | | **Deviations from**  **HDSS campaign information**  **Criterion: information (source)** |
|  | **OPV** | **VAS** | **MV** | **HDSS** | **WHO** | **Rotary #1** |  |
| 08-04-2001 | 0-35 |  |  | X |  |  |  |
| 13-05-2001 | 0-35 |  |  | X | X | X |  |
|  |  | 12-35 |  |  | X |  |  |
| 27-01-2002 | 0-35 |  |  | X | X | X |  |
|  |  | 12-35 |  |  | X |  |  |
| 10-03-2002 | 0-35 |  |  | X | X | X |  |
| 10-08-2002 | 0-35 |  |  |  |  | X |  |
| 14-09-2002 | 0-35 |  |  |  |  | X |  |
| 30-03-2003 | 0-35 |  |  | X | X | X |  |
|  |  | 12-35 |  |  | X |  |  |
| 04-05-2003 | 0-35 |  |  | X | X | X |  |
| 26-01-2004 | 0-35 |  |  | X | X | X | Date of month: 18-01-2004 (WHO, Rotary) |
| 29-02-2004 | 0-35 |  |  | X | X | X |  |
|  |  | 12-35 |  |  | X |  |  |
| 31-10-2004 |  | 12-35 |  |  | X |  |  |
| 16-06-2005 |  | 12-35 |  |  | X |  |  |
| 05-12-2005 |  | 12-35 |  |  | X |  |  |
| 25-02-2006 |  |  | 9-35 |  | X |  |  |
| 16-04-2006 | 0-35 |  |  | X | X | X |  |
| 13-05-2006 | 0-35 |  |  | X | X | X |  |
| 11-06-2006 | 0-35 |  |  | X | X | X |  |
|  |  | 12-35 |  |  | X |  |  |
| 06-08-2006 | 0-35 |  |  | X | X | X |  |
| 14-10-2006 |  |  | 9-35 | X |  |  |  |
| 25-11-2006 | 0-35 |  |  | X | X | X |  |
|  |  | 12-35 |  |  | X |  |  |
| 23-12-2006 | 0-35 |  |  | X | X | X |  |
| 03-03-2007 | 0-35 |  |  | X | X | X |  |
| 01-04-2007 |  | 12-35 |  |  | X |  |  |
| 08-04-2007 | 0-35 |  |  | X | X | X |  |
| 01-10-2007 |  | 12-35 |  |  | X |  |  |
| 27-10-2007 | 0-35 |  |  | X | X | X |  |
| 08-12-2007 | 0-35 |  |  | X | X | X |  |
| 03-01-2008 | 0-35 |  |  |  | X | X |  |
| 10-05-2008 |  | 12-35 |  |  | X |  |  |
| 06-06-2008 |  | 6-35 |  | X |  |  |  |
| 29-11-2008 | 0-35 |  |  | X | X | X |  |
|  |  | 12-35 |  |  | X |  |  |
| 03-01-2009 | 0-35 |  |  | X | X | X |  |
| 06-06-2009 |  | 6-35 |  | X | X |  | Age group: 12-35 months (WHO) |
| 10-01-2010 | 0-35 |  |  | X | X | X |  |
| 14-02-2010 | 0-35 |  |  | X |  | X |  |
|  |  | 12-35 |  |  | X |  |  |
|  |  |  | 9-35 |  | X |  |  |
| 29-05-2010 |  | 6-35 |  | X | X |  | Age group: 12-35 months (WHO) |
| 08-01-2011 | 0-35 |  |  | X | X | X |  |
|  |  | 12-35 |  |  | X |  |  |
| 12-02-2011 | 0-35 |  |  | X | X | X |  |
| 29-05-2011 |  | 12-35 |  |  | X |  |  |
| 29-06-2011 |  | 0-35 |  | X |  |  |  |
| 07-01-2012 | 0-35 |  |  | X |  | X |  |
|  |  | 0-35 |  | X |  |  |  |
| 11-02-2012 | 0-35 |  |  | X | X | X |  |
| 12-03-2013 |  | 0-35 |  | X | X |  | Age group: 6-35 months (WHO)  Extent: subnational but including the Chakaria study area (WHO) |
| 02-06-2013 |  | 0-35 |  | X |  |  |  |
| 03-10-2013 |  | 0-35 |  | X | X |  | Date of month: 05-10-2013 (WHO) Age group: 6-35 months (WHO)  Extent: subnational but including the Chakaria study area (WHO) |
| 21-12-2013 | 0-35 |  |  | X |  | X |  |
| 25-01-2014 | 9-35 |  |  | X |  |  |  |
|  |  |  | 9-35 | X | X |  |  |
| 05-04-2014 |  | 6-35 |  | X |  |  |  |
| 25-04-2015 |  | 6-35 |  | X |  |  |  |
| 14-11-2015 |  | 6-35 |  | X |  |  |  |
| 23-01-2016 | 0-35 |  |  |  |  | X | Extent: Subnational but including the Chakaria study area |
| 20-02-2016 | 0-35 |  |  |  |  | X | Extent: Subnational but including the Chakaria study area |
| 16-07-2016 |  | 6-35 |  | X |  |  |  |
| 10-12-2016 |  | 6-35 |  | X |  |  |  |
| 29-04-2017 |  |  | 9-35 | X | X |  | Extent: subnational but including the Chakaria study area (WHO) |

#1 Rotary only had information on OPV campaigns. Grey shading marks campaigns for other intervention types than OPV for which Rotary had no information.

**Supplementary Table 3. Overview of measles vaccination (MV) campaign information from two different sources: health and demographic surveillance system (HDSS) and the World Health Organisation (WHO). Reference MV campaign information is from the HDSS. Number of fully matched, partially matched and extra additional non-matched MV campaigns.**

|  | **Guinea-Bissau, Bandim Health Project HDSS** | | **Bangladesh, Chakaria HDSS** | |
| --- | --- | --- | --- | --- |
|  | **Number of MV campaigns** | **% follow-up time**  **matched with HDSS MV campaigns exposure**  **(after; before)** | **Number of MV campaigns** | **% follow-up time**  **matched with HDSS MV campaigns exposure**  **(after; before)** |
| Complete | 3 | (100; 100) | 2 | (100; 65.0) |
| Extra MV campaigns in WHO data | 0 | N/A | 2 | (96.9; 82.9) |
| **Total number of MV campaigns in source** | 3 | (100; 100) | 4 | (97.8; 82.9) |
| Extra MV campaigns in HDSS data | 0 | N/A | 1 | (97.8; 100) |
| **Total number of MV campaigns in HDSS** | 3 |  | 3 |  |

**Supplementary Figure 1. Deviation in HR for the effect of OPV campaigns on mortality based on proportion of time matched by intervention type. Linear regression estimates with absolute deviation of HR as a function of proportion of time after OPV campaigns not matched, presented with estimated linear equation and R-squared values.
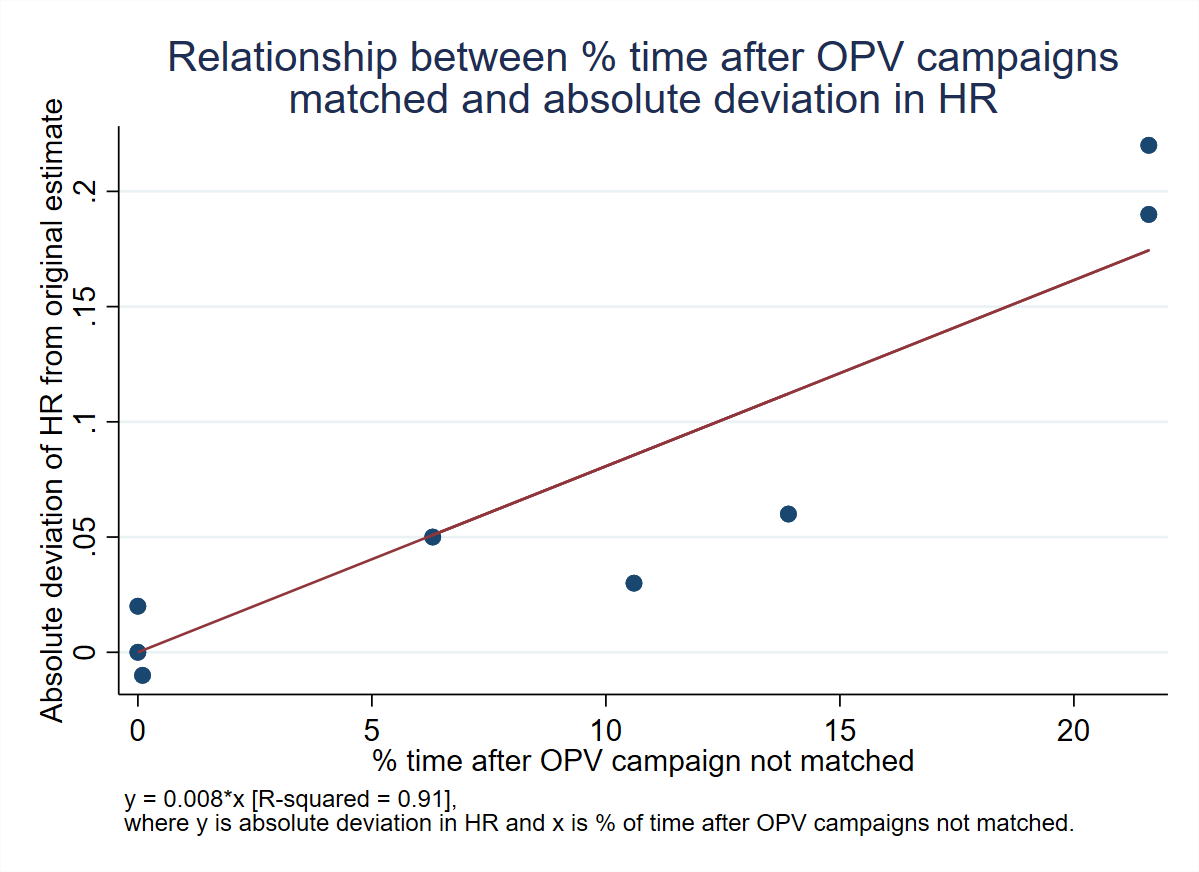
**


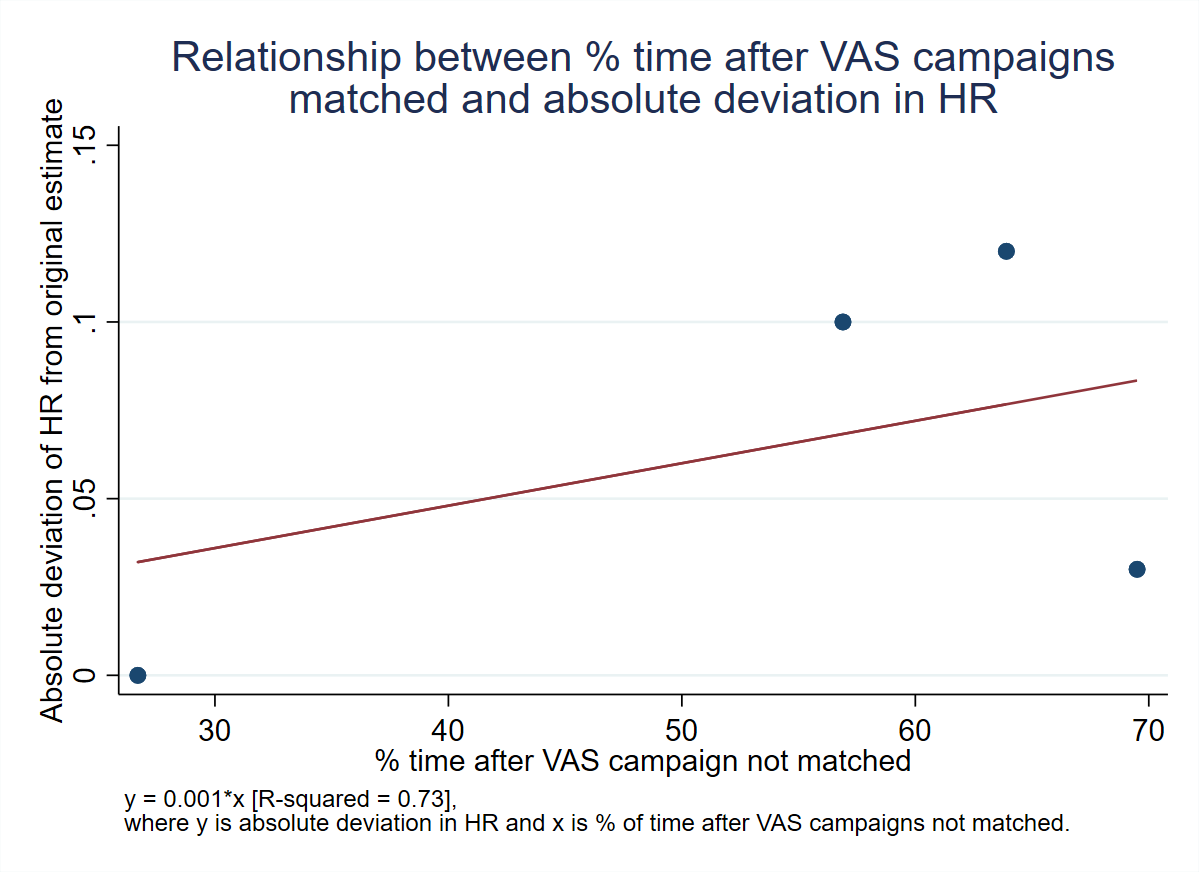


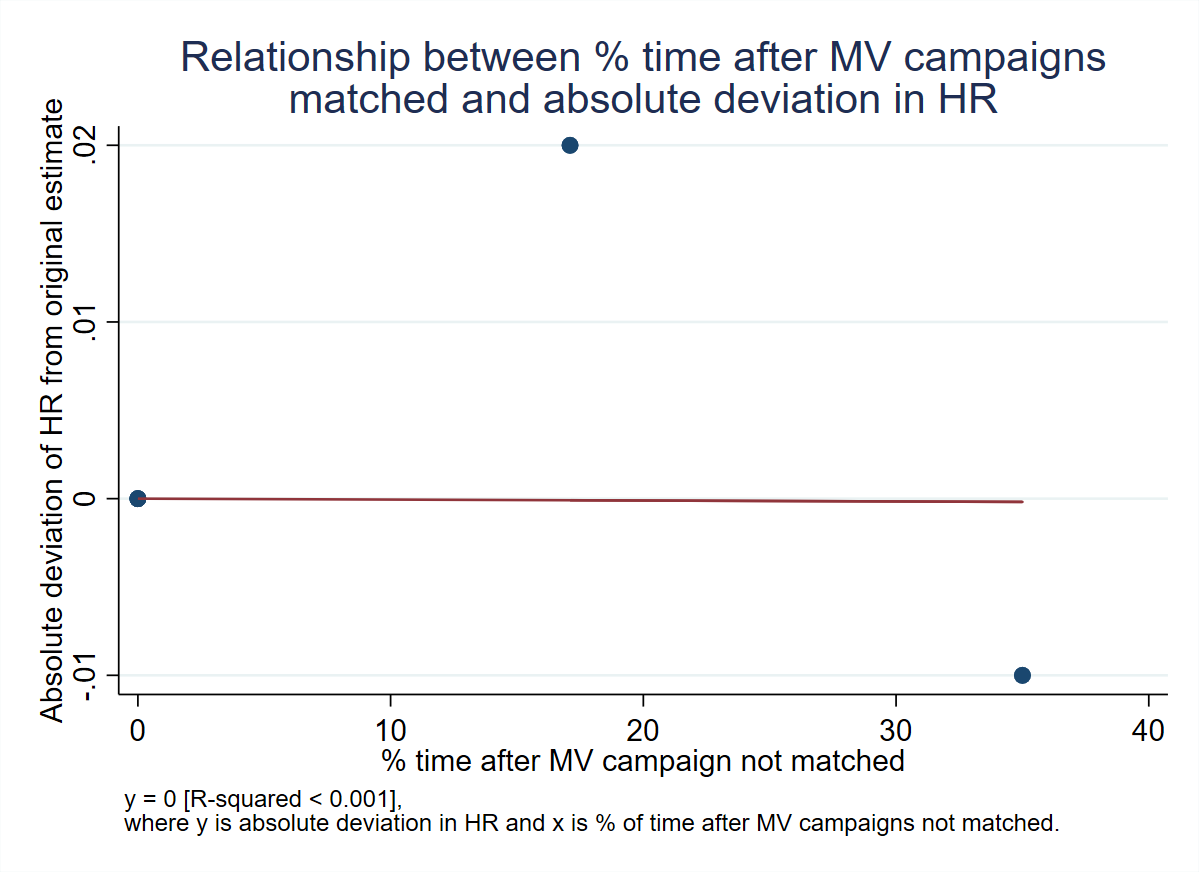


**Supplementary Figure 2. Overview of oral polio vaccination (OPV) campaigns alone or administered with other interventions conducted in Guinea-Bissau and Bangladesh by campaign information source over calendar time. Each “+” represents a national campaign.**
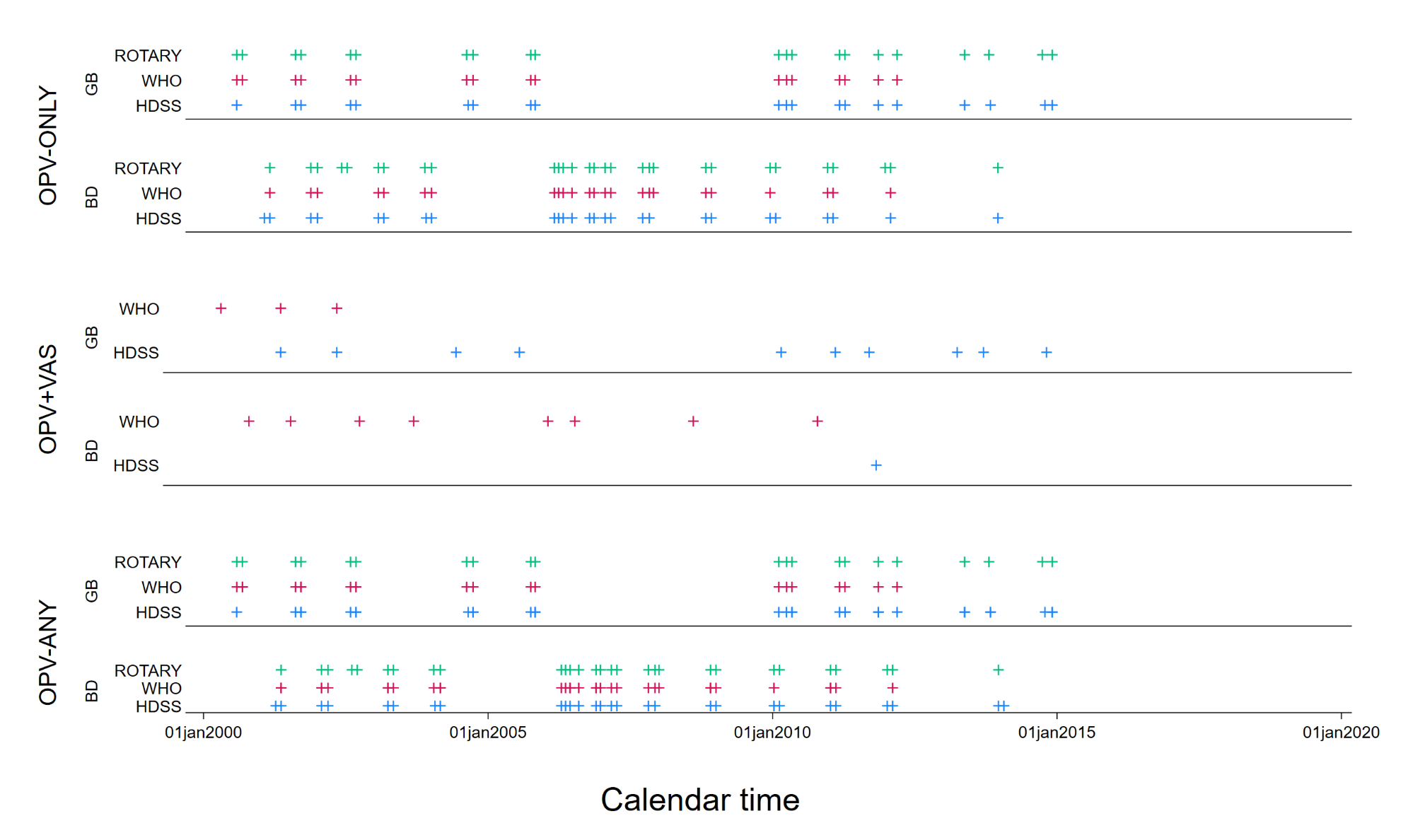


***GB=Guinea-Bissau, BD=Bangladesh, ROTARY=Rotary international, WHO=World Health Organization, HDSS=Health and Demographic Surveillance System.***

**Supplementary Figure 3. Overview of vitamin A supplementation (VAS) campaigns alone or administered with other interventions conducted in Guinea-Bissau and Bangladesh by campaign information source over calendar time. Each “+” represents a national campaign.**
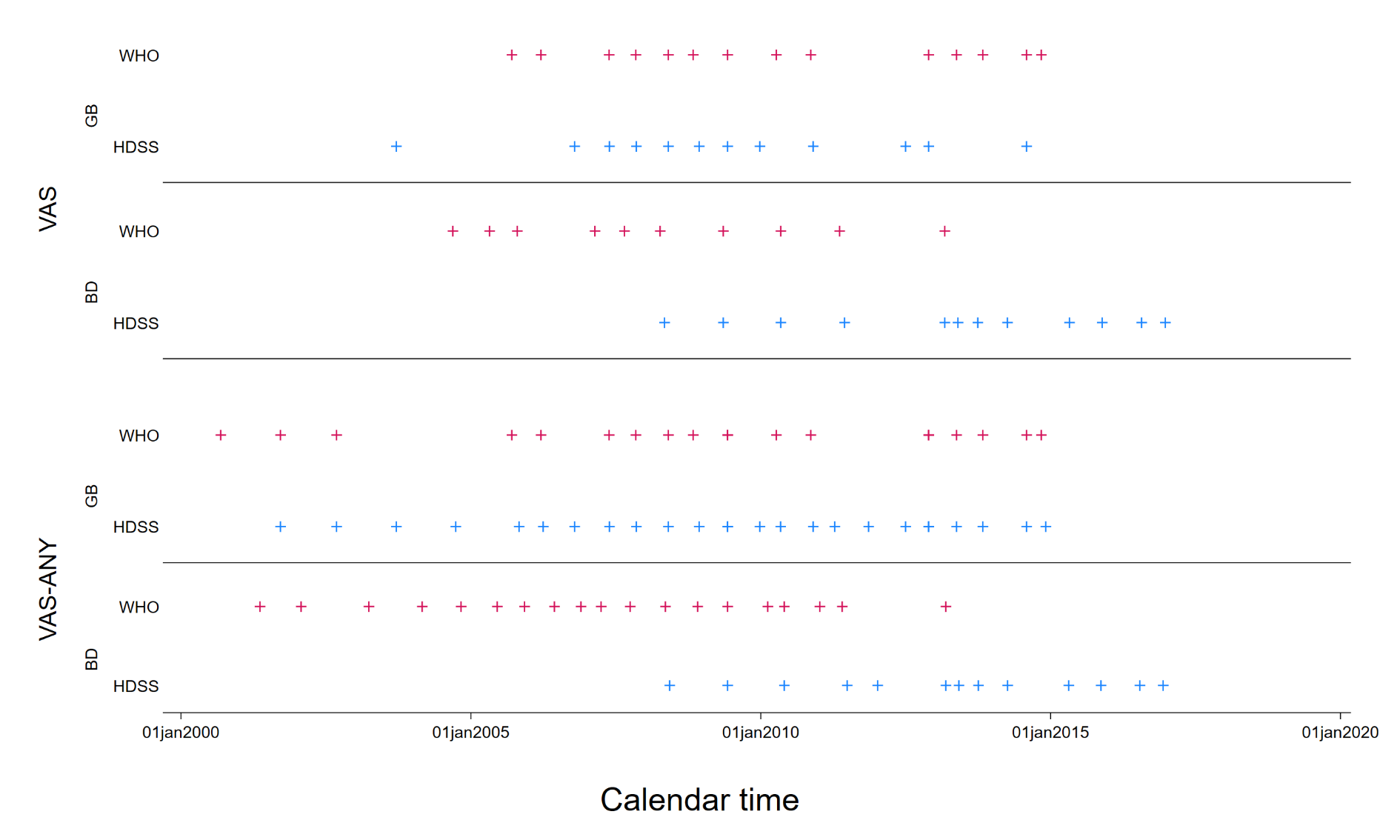


***GB=Guinea-Bissau, BD=Bangladesh, ROTARY=Rotary international, WHO=World Health Organization, HDSS=Health and Demographic Surveillance System.***
